# Tableting of mini-tablets in comparison with conventionally sized tablets: A comparison of tableting properties and tablet dimensions

**DOI:** 10.1016/j.ijpx.2020.100061

**Published:** 2020-11-19

**Authors:** Ard Lura, Guillaume Tardy, Peter Kleinebudde, Jörg Breitkreutz

**Affiliations:** a Institute of Pharmaceutics and Biopharmaceutics, Heinrich Heine University, Universitaetsstr. 1, 40225 Duesseldorf, Germany; b Medel'Pharm, 615 rue du chat botté, Z.A.C. des Malettes, 01700 Beynost, France

**Keywords:** Mini-tablets, Tablets, Tablet properties, Functionalized excipients, Direct compression, Compaction simulator

## Abstract

Mini-tablets are solid dosage forms with increasing interest for pharmaceutical industry due to clinical and biopharmaceutical benefits. But technological aspects on mini-tableting are not fully investigated. Therefore, the impact of punch size and tableting pressure for industrially relevant excipients like microcrystalline cellulose, lactose, isomalt and Ludiflash® are investigated using 8 and 11.28 mm punches for conventionally sized tablets and 1,2 and 3 mm punches for mini-tablets. For evaluation of the effect of tablet size on deformation behaviour and mechanical properties, compressibility, compactibility and tabletability plots are created and evaluated. Deformation behaviour is analysed by *In-Die* Heckel plot and modified Weibull function. Further, specific plastic energy (SPE) profiles are generated out of force-displacement plots. The effect of the adjustment of the aspect ratio towards 1 as in conventionally sized tablets on deformation behaviour and tabletability is analysed. The effect of tablet size on deformation behaviour mainly showed lower yield pressures for conventionally sized tablets, whereas comparable SPEs were obtained with all tablet sizes. Furthermore, mini-tablets indicate better compactibility, as (depending on the excipient) higher tensile strengths were obtained at lower solid fractions. However, no superior tabletability properties are obtained for mini-tablets compared to conventionally sized tablets.

## Highlights

• Mini-tablets show higher compactibility compared to conventionally sized tablets.
• Mini-tablets show specific plastic energy profiles similar to conventional tablets.
• There is no trend for better tabletability for mini-tablets or conventionally sized tablets.
• Highest yield pressures are obtained with mini-tablets.

## Graphical abstract

**Figure:**
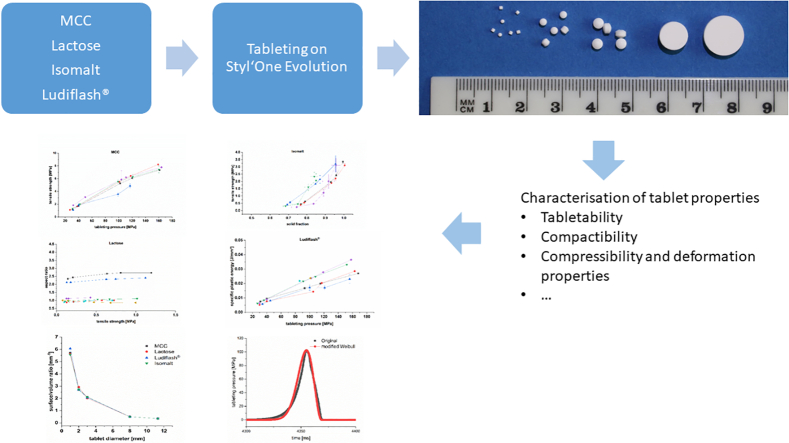

## 1 Introduction

Tablets are the most common used oral drug delivery system. The European Pharmacopeia describes tablets as solid dosage forms produced by compression of a definite volume of powder or granule or by another suitable method. Furthermore, tablets are classified in the Pharmacopeia in different groups according to their manufacturing steps (coated and uncoated tablets etc.) or physico-chemical/biopharmaceutical behaviour (effervescent tablets, gastro-resistant tablets etc.) (Ph.Eur. 10, 2019). An additional classification of tablets can be made according to their size and their resulting scope of application.

Tablets can be further classified into mini-tablets and conventionally sized tablets. Mini-tablets are tablets with a defined size of 2–3 mm (Lennartz and Mielck, 1998). For industrial purpose mini-tablets are produced using conventional tablet presses and multi-tip punches. The first tooling system for the production of mini-tablets was patented by Hershberg (Hershberg, 1965) and since then several suppliers started offering custom tailored tooling systems for mini-tableting. The acceptability and swallowability of mini-tablets as a single or multiparticulate dosage form for children and paediatric patients was proven in several studies (Klingmann et al., 2013; Klingmann et al., 2015; Klingmann et al., 2018). Furthermore, the ability of dose flexibility of different mini-tablet sizes and different drug loading was shown by producing mini-tablets by direct compression or an intermediate high shear granulation step (Gupta et al., 2020; Mitra et al., 2020). Mini-tablets are therefore considered a promising dosage form for pharmaceutical companies for developing child-appropriate medicines.

From the technological point of view, the so far investigated benefit of manufacturing mini-tablets is their advantage compared to pellets. They are highly reproducible in size and weight. Further, a smoother surface compared to pellets lead to a more even coating process due to less coating time and material with lower loss of mass resulting in lower overall costs and higher product stability (Gaber et al., 2015; Munday, 1994). However, the benefit of producing mini-tablets instead of conventionally sized tablet has not been completely investigated yet. Studies and observations indicate better tabletability or compactibility of mini-tablets. A formulation containing 99.5% pankreatin was developed by Pich and Moest. It was not possible to perform tableting with 10 mm punches as the produced tablets showed low mechanical stability and were not suitable for further manufacturing steps. By reducing the tablet size to 2.25 mm, the tableting of the formulation was feasible and was patented by the company Nordmark (Pich and Moest, 1984). Lennartz and Mielck showed that the capping tendency of paracetamol-lactose mixtures could be reduced with decreasing tablet size, as more mechanically stable tablets were obtained with high amounts of paracetamol compared to tablets with 5 mm size (Lennartz and Mielck, 1998). These observations indicate that the mechanical properties of poorly tabletable entities may be improved by producing mini-tablets.

The various phases during tableting are well described. First, particles undergo a rearrangement phase, where the particles or granules are rearranged within the die upon first contact with the (upper) punch and start volume reduction. Afterwards, depending on the material properties, elastic deformation, plastic deformation and fragmentation of the particles occur during compression at increased solid fraction to form a mechanical solid compact. During decompression, as the punch displacement becomes larger, mainly elastic recovery occurs. The last stage is the ejection of the tablet by an upward movement of the lower punch. The stages of deformation are not following a strict order but occur simultaneously.

The mechanical properties of the tablet are not only influenced by the material properties, but also by tablet geometry and size, as compression pressure distribution and resulting tensile strengths are affected by the size and tooling (Amidon et al., 2014; Çelik and Driscoll, 1993; Eiliazadeh et al., 2004; Hiestand et al., 1977; Osamura et al., 2017; Sixsmith and McClueskey, 1981; Train, 1956; York, 1978). According to the activation energy theory, smaller tablets show higher mechanical activation and therefore higher mechanical strength (Hüttenrauch et al., 1985; Jacob and Huettenrauch, 1982). Additionally, Lammens et al. showed for 11 to 17 mm lactose tablets a decrease in tableting pressure for achieving certain tablet densities (Lammens et al., 1980). There are also non-pharmaceutical material investigations on the effect of tablet size on mechanical strength. Kennerly et al. showed a decrease in mechanical strength with decreasing dimensions for sodium chloride tablets (Kennerley et al., 1977) and Ryshkewitz and Duckworth showed an increase in strength of ceramics with decreasing size at the same porosity (Duckworth, 1953; Ryshkewitch, 1953). But these statements were made with tablets, which cannot be classified as mini-tablets.

This study systematically evaluates the influence of tablet dimensions on mechanical properties using the tablet press Styl'One Evolution. Styl'One Evolution is a fully instrumented compaction simulator, which can be used especially for research and development purposes. Contrary to previous investigations with rotary, eccentric or hand-lever presses it is possible to conduct highly precise tableting experiments under defined conditions with a variety of tooling systems and tableting possibilities as well as minimal material consumption. A further aim of this study is to compare Heckel equation (Heckel, 1961), modified Weibull function (Konkel and Mielck, 1997) and energy profiles out of force-displacement plots, with respect to the informative value regarding the deformation behaviour and plastic energy. The tableting process was conducted with pharmaceutically relevant excipients and without the influence of an active pharmaceutical ingredient (API). Frequently used excipients like microcrystalline cellulose (MCC), which is known for mainly plastic deformation (David and Augsburger, 1977) and agglomerated α-lactose (Tablettose® 80), which is described as a more brittle material (Lerk, 1993) are investigated. Isomalt and Ludiflash® gained a certain relevance in formulation development especially for mini-tablets for paediatric use after recent publication of feasibility and clinical studies (Bajcetic et al., 2019; Lura et al., 2019). Functionalized isomalt (galenIQ™721) mainly undergoes plastic deformation whereas d-mannitol (co-processed in Ludiflash®) has more brittle properties (BASF, 2012; Bolhuis et al., 2009; Wu and Sun, 2007). Differences during tableting should be seen regarding deformation behaviour and tableting properties. In this study the terms *compressibility* (solid fraction vs. pressure), *compactibility* (tensile strength vs. solid fraction) and *tabletability* (tensile strength vs. pressure) are used (Amidon et al., 2014).

## 2 Materials and methods

### 2.1 Materials

Four different excipients were investigated. Microcrystalline cellulose (Vivapur 102, JRS Pharma, Germany), lactose (Tablettose® 80, Meggle, Germany), isomalt (galenIQ™721, BENEO-Palatinit, Germany) and Ludiflash®, a co-processed excipient based on d-Mannitol, crospovidone and a polymer dispersion of polyvinyl acetate (BASF, Germany). External lubrication was applied using magnesium stearate (Parteck® LUB MST, Merck, Germany) or sodium stearyl fumarate (PRUV®, JRS Pharma, Germany).

### 2.2 Methods

#### 2.2.1 Powder density

Apparent density of the excipients was measured in triplicate using helium pycnometry (AccuPyc 1330, Micromeritics, Norcross).

#### 2.2.2 Particle size

The particle sizes of MCC, lactose, isomalt and Ludiflash® were measured in triplicate using laser diffraction (Mastersizer 3000, Malvern, UK) at an air pressure of 0.8 bar.

#### 2.2.3 Flow properties

To evaluate the flow properties of the used excipients, angle of repose, Hausner ratio and flow rate through an orifice (orifice diameter: 1.5 cm and 50 g sample) were analysed in triplicate according to Ph.Eur.

#### 2.2.4 Tableting

The materials were tableted under standard conditions (24 °C / 40% RH) on the tablet press Styl'One Evolution (Medel'Pharm, France) with a constant machine speed of 20%, respectively 10% for 2 and 3 mm single tips in one compression mode at constant filling heights for each excipient and tablet size. For mini-tablets, 1 and 2 mm concave EU-B 19-tip tooling (Ritter Pharma, Germany) and EU-D 3 mm concave 19-tip tooling, respectively EU-B 2 and 3 mm single tips (Natoli-Engineering, USA) were used and manual die filling was performed. The consistency of manual die filling was monitored with weight control after every 5th or 10th tableting cycle. Conventionally sized tablets were produced using EU-B 8 mm (Ritter Pharma, Germany) and EU-B 11.28 mm (Natoli Engineering, USA) flat faced punches. For 8 and 11.28 mm tablets a forced feeder was used during production. External lubrication with magnesium stearate (MgSt), respectively for 2–8 mm Ludiflash® tablets sodium stearyl fumarate (SSF), was applied. MgSt did not show a sufficient lubrication effect for Ludiflash® mini- tablets, as even at low tableting pressure high ejection forces occurred. All tablets were produced at five different tableting pressures (30, 40, 100, 120, 160 MPa). The pressures were set to these values for security purposes of the mini-tablet tooling and for working in a range of realistic manufacturing conditions. To evaluate the impact of the aspect ratio of the tablets, the conventionally sized tablets (8 and 11.28 mm) were also compressed at the highest possible filling level and the same tableting pressures aiming at an aspect ratio (AR) (diameter/height) close to 1.

#### 2.2.5 Compressibility

##### 2.2.5.1 Heckel plot

The Heckel equation describes the compressibility of a material as a function of loss of porosity during pressure following 1st order kinetics (Heckel, 1961).

(1) $$-\frac{\mathrm{d\varepsilon}}{\mathrm{dp}}=k*\varepsilon$$

(2) $$\text{Respectively}:\ln\left(\frac{1}{\varepsilon }\right)=k*p+A$$

ɛ = Porosity [%]
p = Pressure [MPa]
k = Constant
A = Constant

The linear part of the curve should express the plastic deformation of a material. The reciprocal value of the slope expressed as the mean yield pressure (P_y_) indicates the plasticity of the material (Hersey and Rees, 1971). Values below 80 MPa designate a low resistance against deformation and therefore characterize more plastic deformation behaviour than P_y_ above 80 MPa (Duberg and Nyström, 1982; York, 1992). *In-die* Heckel-plot was determined using the Analis Software (Medel'Pharm, France) of Styl'One Evolution. A tableting pressure of about 100 MPa was applied for evaluation and comparison of the *in- die* Heckel plots for all excipients and punch sizes using multi-tip tooling for the mini-tablets. The negative natural logarithm of the porosity was plotted against the tableting pressure of one tableting cycle and the yield pressure was calculated from the linear part of the plot. A coefficient of determination was set to at least 0.99. For calculation of means and 95% confidence intervals six cycles were determined for each excipient and punch size.

##### 2.2.5.2 Modified Weibull function

The analysis of force-time plots can reveal information about the deformation behaviour of excipients. As the force-time plots are sensitive to tableting speed, the speed of Styl'One Evolution was kept constant at 20% for all tableting experiments, except for mini-tableting with single-tip punches. The Weibull function was used and modified by Dietrich and Mielck (1984) and simplified by Konkel and Mielck (1997) to two parameters β and γ to describe the deformation behaviour of excipients following these two equations:

(3) $$P\left(t\right)=P_{\mathrm{UP},\max}*{\left(\frac{t_{\mathrm{end}}-t}{t_{\mathrm{end}}-t_{\max}}\right)}^{\gamma }*{e^{1-\left(\frac{t_{\mathrm{end}}-t}{t_{\mathrm{end}-t_{\max}}}\right)}}^{\gamma }$$

(4) $$\beta =\frac{t_{\mathrm{end}}-t_{\max}}{t_{\mathrm{end}}-t_{\mathrm{start}}}*100$$

P(t): Pressure at time t
P_UP,max_: Maximal pressure on upper punch
t_start_: First applied pressure point (where p exceeds >1 MPa)
t_end_: Last applied pressure point (where p falls below <1.5 MPa)
t_max_: Time at maximal pressure

Parameter γ describes the symmetry of the force-time curve and indicates the resistance against densification of a material. High values for γ correspond to materials with elastic and brittle properties. Parameter β describes the time-dependent position of maximum pressure. High values for both parameters indicate elastic and brittle deformation behaviour and low values plastic deformation behaviour (Dietrich and Mielck, 1984). For each excipient at each tableting pressure three tableting cycles were chosen randomly and the parameters were calculated and presented in β/ γ diagrams (Haaks, 1988).

#### 2.2.6 Plastic energy

The energy profiles were measured during the tableting process with Styl'One Evolution and Analis Software. The plastic energy is defined as the area between the compression and decompression curve. For calculation of the plastic energy, the means of all tableting cycles (10–40 cycles) for each tableting pressure were included and not only of one compression cycle. In order to compare the energy profiles of the tablets including the different dimensions, the specific energy density was calculated comprising the energy in proportion to the volume. The resulting areas under the curve for plastic energy are calculated from force-displacement curves. The specific energy density (SPE; Eq. (5)) has therefore to be adjusted to the total volume of tablets, produced in one cycle. For example, 19 mini-tablets are produced with one compression cycle due to the 19-tip tooling. From this cycle a force-displacement plot is created with a certain force to achieve a comparable tableting pressure for all mini-tablets and conventionally sized tablets. Therefore, it is mandatory to include for calculation of an energy density the total volume. In this case the total volume of 19 mini-tablets and for a single tip the volume of one tablet. Following equation was used:

(5) $$\omega =\frac{E}{V}$$

ω = Specific energy density [J/mm^3^]
E = Measured energy [J]
V = Volume all tablets produced with one compression cycle [mm^3^].

Volume of flat faced tablets was calculated with following equation:

(6) $$V=\pi *r^{2}*h$$

Volume of convex tablets was calculated with following equations.

1. Cap volume (V_cap_)

(7) $$\mathrm{Vcap}=\frac{\pi }{6\;}*h\left(3*r^{2}*\mathrm{cap}\;\mathrm{heigh}t^{2}\right)$$

2. Cylindrical volume (V_cyl_)

(8) $$\mathrm{Vcyl}=\pi *r^{2}*h$$

3. Volume of convex tablet (V_con_)

(9) Vcon=Vcyl+2∗Vcap

The specific energy density, respectively the specific plastic energy (SPE) is plotted against the tableting pressure.

#### 2.2.7 Compactibility

For the evaluation of the compactibility of the tablets, tensile strengths (TS) were plotted against the solid fraction (SF) of the tablet. SF was calculated with the following equation:

(10) $$\mathrm{SF}=\frac{\rho \;\left(\mathrm{tablet}\right)\;\left[\frac{\mathrm{mg}}{mm^{3}}\right]}{\rho \;\left(\mathrm{powder}\right)\left[\frac{\mathrm{mg}}{\mathrm{mm}3}\right]}$$

The density ρ of the tablet was calculated with the following equation:

(11) $$\rho \;\left(\mathrm{tablet}\right)=\frac{\mathrm{weight}\;\left[\mathrm{mg}\right]}{\mathrm{volume}\;\left[mm^{3}\right]}$$

The density of the powder was determined as described in Section 2.2.1.

Volume of the tablets was calculated as described in Section 2.2.4.

The weight of 8 and 11.28 mm tablets was measured using SmartTest ST 50 (Sotax, Switzerland). The weight of 2 and 3 mm mini-tablets was measured using analytical balance MC 210 P (Sartorius, Germany) and for 1 mm mini-tablets XP56 (Mettler Toledo, USA).

#### 2.2.8 Tabletability

All tablets were stored at least for 24 h under climatic conditions (24 °C/40% RH). The breaking force of the mini-tablets was determined using Texture Analyser XT.plus (Stable Micro Systems, UK) with a pre-speed of 0.1 mm/s and a 0.5 cm flat punch. For determination of the breaking force for 8 and 11.28 mm tablets SmartTest ST 50 (Sotax, Switzerland) was used. Tensile strengths (TS) were calculated using the equation of Fell and Newton (1970). This equation is used for flat faced tablets. Pitt and Newton investigated the tensile fracture of biconvex cylindrical discs with a central cylinder thickness to diameter ratio of 0.06 to 0.3 and proposed another equation (Pitt and Newton, 1988). Mini-tablets have a ratio above 0.3, as they show a nearly spherical shape in most cases. Several authors therefore apply the equation of Fell and Newton to convex mini-tablets (Flemming and Mielck, 1995; Lennartz and Mielck, 1998; Tissen et al., 2011). However, in this study tensile strength should also be determined using the equation of Pitt and Newton and compared to the calculated tensile strengths with the equation of Fell and Newton. TS of 3 mm MCC mini-tablets compressed at 160 MPa could not be determined, as security levels of the loading cell of Texture Analyser were reached. The dimensions of the tablets for calculation of aspect ratio (AR) and tensile strength were determined for 8 and 11.28 mm tablets automatically by Smart Tester. For the 2 and 3 mm mini-tablets, measurements were performed manually using a calliper (Mitutoyo Absolute, Japan). For 1 mm mini-tablets image analysis (Leica Microsystems, UK) was used. Furthermore, AR was calculated from the ratio of diameter and height of the tablet. The calculated tensile strengths are plotted against the AR for evaluating the impact of AR on TS. For the tabletability plots, the TS are plotted against tableting pressure. Linear regressions are conducted to evaluate the slope of the plots.

## 3 Results and discussion

### 3.1 Physical properties of the excipients

The physical characterization of the excipients, which impacts the continuous tableting process, are shown in Table 1.

**Table 1:** Physical characterization of MCC, lactose, isomalt and Ludiflash®. All values: *n* = 3, mean ± SD.

	MCC	Lactose	Isomalt	Ludiflash®
D_10_ [μm]	32.2 ± 0.2	31.8 ± 0.7	35.7 ± 1.1	29.0 ± 1.1
D_50_ [μm]	125.6 ± 1.0	152.9 ± 9.9	175.4 ± 16.2	84.4 ± 6.2
D_90_ [μm]	263.6 ± 2.8	463.3 ± 31.6	404.7 ± 21.4	251.5 ± 47.3
Angle of repose [°]	37.0 ± 1.1	27.5 ± 0.0	30.4 ± 1.3	31.2 ± 1.3
Flow rate through an orifice [s]	–	3.0 ± 0.0	4.0 ± 0.0	5.0 ± 1.0
Hausner ratio	1.3 ± 0.0	1.2 ± 0.0	1.3 ± 0.0	1.2 ± 0.0

Flow properties described as angle of repose and flow rate through an orifice reveal that lactose, isomalt and Ludiflash® show the best flowability and emphasize the suitably of these excipients as fillers/binders for formulation development. MCC however shows the highest angle of repose among the four tested excipients and its flow rate could not be determined as the powder did not flow through the orifice. The Hausner ratio shows comparable results for all excipients and does not indicate a great change between bulk and tapped density, which is an important indicator for a robust tableting process.

### 3.2 Compressibility and deformation properties

#### 3.2.1 Heckel plot evaluation

For many pharmaceutical materials the linear part in the Heckel plot is only reached at high tableting pressures, which have no relevance in tablet manufacturing (Sonnergaard, 1999). Therefore, we performed an *in-*die Heckel plot for tableting pressures, which are commonly used and have more pharmaceutical relevance in tablet manufacturing. For defining an absolute deformation behaviour with the help of yield pressures, the Heckel equation has too many deficits, since among other things it is not a reproducible material constant (Sonnergaard, 1999). Nevertheless, as the data were obtained under similar conditions, it can be used for the purpose of describing the development of the yield pressure within the same excipient under variation of the tablet size and dimension, but no general statement should be made about the deformation behaviour itself. The results for the yield pressure obtained by *in-die* Heckel plot show differences between the excipients and tablet dimensions (Table 2). Not all yield pressures of the four tested excipients show a clear shift of lower yield pressures towards the mini-tablets. On the contrary, tablets with conventional sizes show comparable or even lower yield pressures for the tested excipients.

**Table 2:** Calculated yield pressures (*P*_*y*_) in MPa from *in- die* Heckel plot for MCC, lactose, isomalt and Ludiflash® and adjusted aspect ratios (AR) represented as mean values ± SD and upper and lower 95% confidence level (UCL and LCL); *n* = 6 per tablet size.

MCC			
Tablet size [mm]	*P*_*y*_ ± SD	LCL _*95%*_	UCL _*95%*_
1	92.8 ± 16.5	75.5	110.2
2	64.3 ± 6.1	57.9	70.8
3	135.2 ± 8.1	126.7	143.6
8	53.3 ± 1.8	51.5	55.2
11.28	52.2 ± 4.2	47.7	56.6
8 AR	51.2 ± 4.3	46.7	55.6
11.28 AR	52.5 ± 5.7	46.5	58.5


Lactose			
Tablet size [mm]	*P*_*y*_ ± SD	LCL _*95%*_	UCL _*95%*_
1	192.2 ± 16.7	174.6	209.7
2	174.0 ± 14.3	159.0	189.0
3	199.0	–	–
8	122.5 ± 6.1	116.1	128.9
11.28	120.8 ± 1.5	119.3	122.4
8 AR	126.2 ± 2.2	123.8	128.5
11.28 AR	140.5 ± 5.5	134.7	146.3


Isomalt			
Tablet size [mm]	*P*_*y*_ ± SD	LCL _*95%*_	UCL _*95%*_
1	103.7 ± 15.2	87.7	119.6
2	67.3 ± 6.0	61.1	73.6
3	68.2 ± 6.2	61.6	74.7
8	57.7 ± 0.8	56.8	58.5
11.28	57.8 ± 2.1	55.6	60.1
8 AR	53.3 ± 2.4	50.8	55.9
11.28 AR	55.3 ± 3.2	52.0	58.7


Ludiflash®			
Tablet size [mm]	*P*_*y*_ ± SD	LCL _*95%*_	UCL _*95%*_
1	90.5 ± 17.5	72.1	108.9
2	117.7 ± 16.7	100.2	135.2
3	83.3 ± 26.0	56.0	110.6
8	55.3 ± 1.7	54.0	57.6
11.28	56.5 ± 2.3	54.0	59.0
8 AR	56.8 ± 2.3	54.4	59.3
11.28 AR	57.0 ± 1.4	55.5	58.5

Conventionally sized MCC tablets show yield pressures with lower scattering of standard deviations (SD) and 95% confidence intervals (CI) compared to mini-tablets in the range between 51 and 53 MPa. Within the mini-tablets, there is a variation of yield pressures, as 2 mm mini-tablets have significantly lower yield pressures than 1 and 3 mm tablets and 3 mm tablets show significantly the highest yield pressure of all tablets. The variation of the height for the conventional size tablets towards an adjusted AR has no significant impact on the yield pressure.

Lactose tablets show a comparable trend regarding the yield pressure against the tableting size. There are significant differences between mini-tablets and conventionally sized tablets. Again, 3 mm tablets show the highest resistance against deformation for lactose, but as only one cycle could be performed due to very high ejection forces this is just a trend but no statistic parameters could be calculated.

For isomalt there are no significant differences in yield pressures between the 2 and 3 mm mini-tablets and conventionally sized tablets. Contrary to MCC and lactose, 1 mm mini-tablets show the highest yield pressure. An approximation of AR lead to a non-significant decrease of yield pressure.

Ludiflash® tablets show significant differences between mini-tablets and conventional tablets as well. In this case, 2 mm tablets show the highest yield pressure for Ludiflash®. 8 mm AR and 11.28 mm AR tablets show lower mean values for the yield pressure compared to the conventionally sized tablets, but without a significant impact.

The yield pressures generated by the Heckel equation are highly discussable and must be judged with caution as to their meaningfulness of deformation behaviour. Nevertheless, the comparison of the different tablet sizes showed a significant trend for lower yield pressures towards conventionally sized tablets. For several common excipients like MCC different yield pressures were obtained in the range of 104 MPa (Paronen, 1986), 84.4 MPa (Yu et al., 1989) or 47.6 MPa (Roberts and Rowe, 1987), whereas for lactose yield pressures of 263.1 MPa (Ilić et al., 2009) or between 150 and 200 MPa (Ilkka and Paronen, 1993; Roberts and Rowe, 1987) were obtained. In previous studies a comparable grade of isomalt showed a yield pressure of about 115 MPa (Grote et al., 2019), which is still higher than the obtained yield pressures of this study, although the yield pressures were analysed with Styl'One Evolution. An explanation for a better deformation behaviour of mini-tablets for pharmaceutical excipients during realistic tableting procedures cannot be supported by this data. However, the geometry was changed as different punch geometries were used (flat for conventionally sized tablets and convex for mini-tablets). The main differences between the results within the mini-tablets may therefore be bigger than for flat faced tables. The results for 8 and 11.28 mm are more reproducible as the pressure distribution is more homogenous than in a convex tablet and therefore constant A of the Heckel equation is less affected by the applied pressure than for convex tablets (Eiliazadeh et al., 2004; Sixsmith and McClueskey, 1981; Heckel, 1961).

#### 3.2.2 Modified Weibull function

**Fig. 1:**
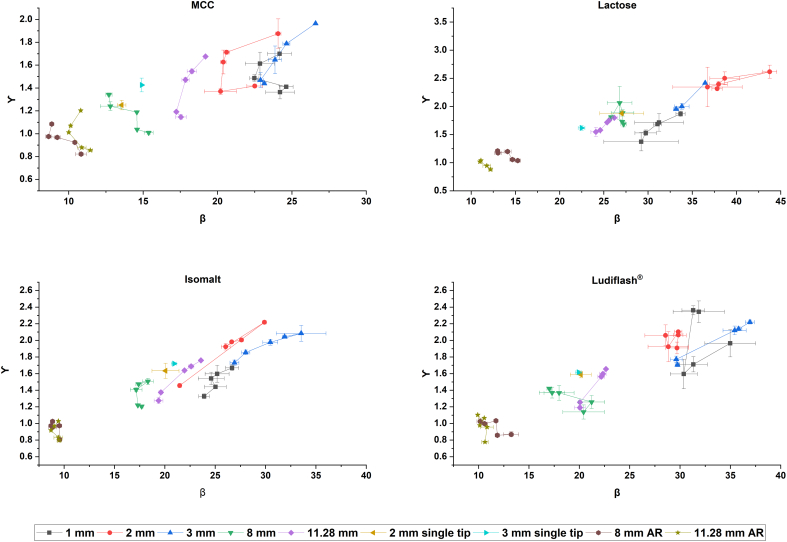
β/γ plots for MCC, lactose, isomalt and Ludiflash® over five tableting pressures and different tablet dimensions including approximated aspect ratio (AR); each tableting pressure: *n* = 3; mean ± SD.

The obtained graphs in Fig. 1 show an excipient-dependent alteration of deformation properties with varying tablet dimension. Relating to Fig. 1 several general results and conclusions can be drawn from the modified Weibull plots. It is noticeable that the results of modified Weibull function are highly dependent on the used excipient, the diameter of the tablet, the applied pressure, the use of single or multi-tip for mini-tablets and the impact of the aspect ratio for conventionally sized tablets. For a better comparison it is mandatory to use identical tableting parameters, so that the shape of the force-time curve depends on the material properties only. In the following, these impact factors are discussed and a conclusion can be drawn for the application of the modified Weibull function.

##### 3.2.2.1 Impact of excipient

The deformation properties of MCC and isomalt show mainly plastic behaviour, whereas lactose and d-mannitol are described as brittle and elastic materials in literature, which is partly confirmed in the Heckel plots in Section 3.1.1. and in literature (Bolhuis et al., 2009; David and Augsburger, 1977; Leane et al., 2015; Sun et al., 2009). The results of modified Weibull for the conventionally sized tablets are similar for all excipients suggesting the same deformation behaviour. The calculated parameters must not be misinterpreted by comparing the excipients with each other. Obviously, the excipients cannot be directly compared in this study, as different filling heights were used during tableting. However, in contrast to MCC and isomalt, the pattern of the plot for lactose and Ludiflash® look more different. Both excipients show higher values for β and γ in general and support the applicability of the modified Weibull function to distinguish between pharmaceutical excipients and their deformation and material properties. Nevertheless, for a valid comparison between excipients all tableting adjustments have to be identical.

##### 3.2.2.2 Tablet diameter and tableting pressure

The influence of the tablet diameter is evident for the results of all substances. By increasing the tablet diameter towards conventionally sized tablets, the indicator for plasticity increases and for brittleness decreases for all excipients. The conventionally sized tablets indicate a more plastic deformation behaviour than the mini-tablets produced with multi-tip punches. However, for almost all excipients the 8 mm tablets designate higher plasticity than the 11.28 mm, except for lactose, as the data points are located in almost the same regions and show depending on the pressure non-significant results. The impact of 1, 2 or 3 mm mini-tablets on deformation behaviour do not show a systematic behaviour. These observations are consistent with the data obtained with Heckel in Section 3.1.1., as also there no mini-tablet diameter stands out either but depends on the excipient. However, fill-weight variability may also lead to a scattering, as high or low filling levels between the tablet diameters impact the results for β and γ. For modified Weibull (depending on the used excipient and applied pressure) 2 mm mini-tablets indicate more plasticity for MCC whereas for lactose and isomalt 1 mm mini-tablets show this trend. For Ludiflash® (depending on the applied pressure) either 1 or 3 mm mini-tablets lead to lower β and γ.

All tablets show a dependency of the tableting pressure on the calculated β and γ values. The trend to higher γ with increasing tableting pressure can be explained with the formula for calculating γ. Certainly, a change of γ impacts the value of β to some extent, which can also be observed in the plots. For some excipients and tablet diameters this effect could be significant, whereas for others it is not. The scattering of the values within the tableting pressures and diameters can be explained with slightly different main pressures in the tableting process. Some deviations in the P_UPmax_ lead to other data points for t_max_, t_start_ and t_end_ resulting in a variance of β and γ. The scattering is also a hint for the precision of tableting aiming a tableting pressure. Obviously, the scattering can be seen especially for more brittle materials like lactose and Ludiflash® in mini-tablets. Nevertheless, for 2 mm Ludiflash® mini-tablets the tableting pressure does not appear to have a significant effect on β and γ values.

##### 3.2.2.3 Impact of single-tip and multi-tip

The mini-tablets produced by multi-tip tooling indicate in the plots for each excipient the least plasticity and a higher scattering of the β and γ values within the tableting pressures. A spot check using a 2 and 3 mm single-tip at similar tableting settings (filling height and target tableting pressure) lead to a decrease of β and γ for all excipients. However, it has to be noted, that the tableting speed for the single-tip was 10% and not 20%. As the speed does have an impact on the symmetry of a pressure-time plot, the results are not comparable at the same level. The difference in calculation especially for β is highly impacted, as the time points are weighted in the formula in the numerator as well as in the denominator. Particularly for more plastic materials like MCC and isomalt, the impact of the type of punching tool on the deformation behaviour has to be evaluated as the main deformation behaviour for more brittle materials like lactose or Ludiflash® should not be time-dependent and the impact of the speed should be minimal (Rees and Rue, 1978). According to the β/γ plot, the use of a single-tip for the mini-tablets direct a shift towards more plasticity. Since neither the size of the tablet nor the tableting conditions are affected by a single-tip, it is questionable how the tooling impacts the results. Under experimental conditions the use of the single-tip proposes highly comparable results with conventionally sized tablets and indicate negligible differences in deformation behaviour depending on the tablet diameter. Nevertheless, we have to consider the fact that the tableting speed has to be adjusted to the same level, as an increase of tableting speed might change the results significantly.

##### 3.2.2.4 Impact of aspect ratio

By adjusting the aspect ratio (AR) of the conventionally sized tablets towards the mini-tablets closer to 1, even lower β and γ values were obtained indicating a very low resistance against deformation at comparable tableting pressures. This observation is similar for all excipients. As the filling height and therefore the masses of the powder are much larger, a compression with a certain tableting pressure may be faster compared to a compaction with lower filling height and therefore lower mass. As the compaction is faster, lower β and γ values are calculated. These observations do not fit to the calculated yield pressures from the *in-die Heckel plot* in Section 3.1.1., as yield pressures are still highly comparable between adjusted AR and original conventionally sized tablets. This observation questions the applicability of the modified Weibull function. The effect of filling height and therefore the effect of the mass may distort the interpretation of the calculated data.

##### 3.2.2.5 Conclusion on modified Weibull function

**Fig. 2:**
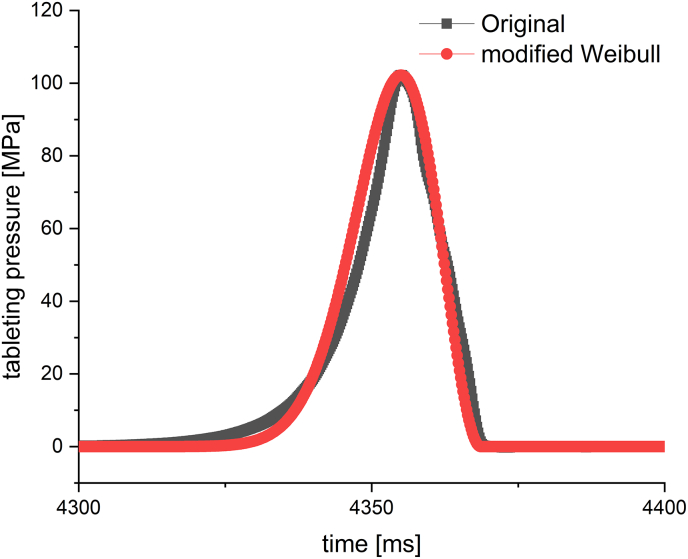
Original pressure-time plot for a 2 mm isomalt tablet and fitted pressure-time plot with modified Weibull function.

The results presented in Fig. 1 provide much information about the applicability of the modified Weibull function. The results of the modified Weibull function, based on a calculation from pressure-time plots, are an interesting approach to define deformation behaviour of materials with only little (pre-experimental) effort. The comparison of the different excipients indicates the applicability of the modified Weibull function, as more plastic materials like MCC show a different signature in the plot compared to a brittle material like lactose. Nevertheless, the modified Weibull function has to be evaluated by comparing different material of same tablet size on similar tablet conditions. However, there are some open questions remaining about the interpretation of the data and the impact of the tabling conditions, as the tooling system of mini-tablets seems to have a great impact on the results. Besides the tooling system, a possible fill-weight variability caused by manual die filling of the mini-tablet produced with a multi-tip obviously lead to a scattering of the data in the β/γ plot. The interpretation of the data regarding a shift of plastic or brittle properties within an excipient depending on the tablet size can be questioned, as the impact of AR shows how sensitive the calculation is on the mass of the tablet. On the other hand, the results definitely coincide with results from the *in-die* Heckel plot regarding a change of deformation behaviour altered by the tablet size. The quality of β and γ depends also on the fit with the modified Weibull function (Fig. 2). If the fit cannot reflect the original pressure-time curve, calculated β and γ parameters would give incorrect information. Furthermore, the susceptibility of pressure-time plots to tableting speed would lead to non-comparable results, if the tableting speed cannot be controlled precisely. Scatterings in pressure above 1, respectively 1.5 MPa before or right after the pressure-time curve would not make a correct calculation possible as well. With respect to Fig. 1 the assessment of the results must be carried out on the base of a relative comparison and not on the base of defining absolute β and γ constants for material properties.

#### 3.2.3 Plastic energy

**Fig. 3:**
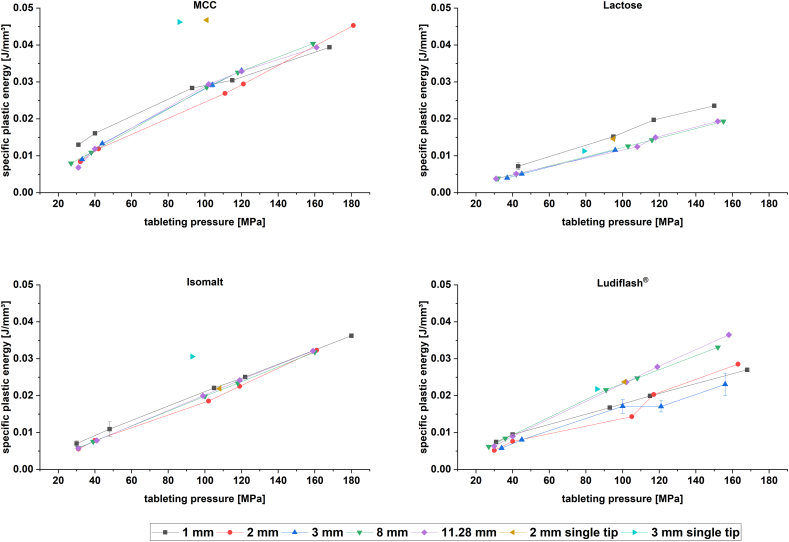
Specific plastic energy plots of MCC, lactose, isomalt and Ludiflash®; *n* = 10–40 tableting cycles; mean ± SD.

For comparison of plastic energies between different tablet sizes, the specific plastic energy (SPE) was calculated according to Eq. (5). This mathematical transformation reveals that the plastic energy per volume is similar for mini-tablets produced with a multi-tip tooling in comparison to conventional tablets (Fig. 3). The plot of SPE vs. tableting pressure shows similar profiles for all excipients. All tablet sizes show an almost linear increase of SPE with higher tableting pressures for all excipients. However, the slopes for MCC and isomalt are higher than for Ludiflash® and lactose.

A spot check with 2 and 3 mm single tip punches at a tableting pressure of about 100 MPa reveal SPEs, which lie for MCC and isomalt slightly above the values of the others. As all mini-tablets were produced under the same conditions regarding filling height and targeted tableting pressure, no major differences should occur in the SPE plot. The effect of the number of tips on SPEs should be evaluated more systematically in a future study.

The hypothesis that materials generally show more pronounced plastic properties, when they are compacted as mini-tablets can be rejected according to this data. These results go hand in hand with the observations made from Heckel plot evaluation (Section 3.2.1) and modified Weibull function (Section 3.2.2).

### 3.3 Compactibility

**Fig. 4:**
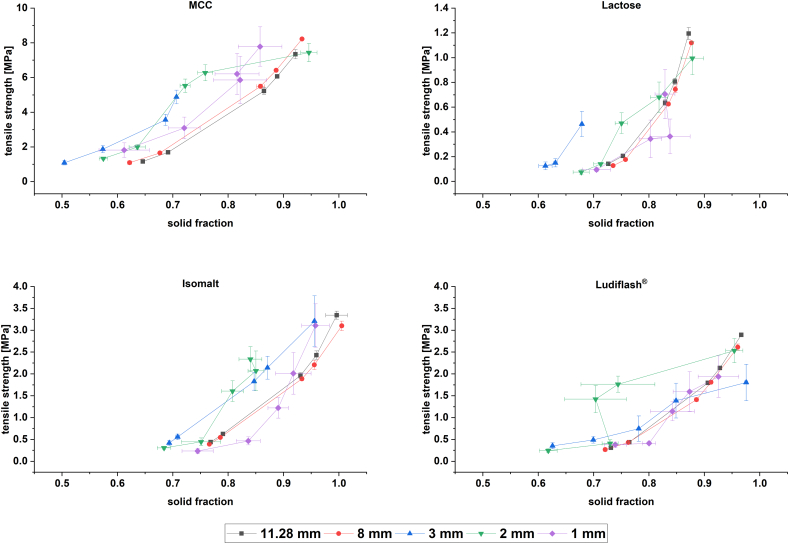
Compactibility plots of MCC, lactose, isomalt and Ludiflash® at different tablet sizes; n = 10; mean ± CI (95% confidence interval).

The compactibility plots (Fig. 4) show the dependence of tensile strenghts (TS) on the solid fraction (SF) for all excipients and tablet sizes. The plots reveal that there are differences in compactibility within tablet sizes. For all excipients the course of the plots for 8 and 11.28 mm look similar, but exhibit different results regarding compactibility properties between the excipients. There are only minor differences in compactibility between 8 mm and 11.28 mm tablets within the same excipient. Major differences can be observed for the plots and the courses of the mini-tablets and excipients. However, in most cases and for all excipients, 2 and 3 mm mini-tablets show better compactibility profiles compared to conventionally sized 8 and 11.28 mm tablets. Obviously, the SF could not be determined precisely especially for 1 mm mini-tablets as the confidence intervals for SF (CI_SF_) scatter most and show insignificant results for most excepients. Therfore, the interpretation of the data for 1 mm mini-tablets is difficult.

The compactibility plot of MCC shows that higher TS can be obtained with mini-tableting at given SF. Even at SF between 0.5 and 0.6 suitable TS between 1 and 2 MPa are reached with all mini-tablet sizes, whereas for conventionally sized tablets a SF of 0.7 is necessarry. Only for a TS of about 7 MPa the SF of 2 mm mini-tablet is higher than for 8 and 11.28 mm. Focusing on the mini-tablets there is a trend noticable as well. The lowest SF with the highest TS are obtained with 3 mm mini-tablets, followed by 2 and 1 mm mini-tablets. However, the fifth TS for 3 mm mini-tablet could not be determined as the overload limit of Texture Analyser was reached. By increasing the SF for 1 mm mini-tablets we cannot make a valid statement on compactibility as almost all CI_SF_ overlap.

The trend of higher compactibility for mini-tablets cannot be highlighted clearly in the compactibility plot of lactose. Nevertheless, the obtained data points with 3 mm tablets show the highest TS with the lowest SF, followed by 2 mm mini-tablets to a TS up to 0.8 MPa. Mini-tablets do not seem to have better compactiblity properties for lactose by producing 1 mm tablets, as a SF between 0.8 and 0.9 is required to achieve a TS up to 0.7 MPa. Nevertheless, the course of 1 mm mini-tablets is not as clear as for the other tablets, as CI_SF_ scatter the most.

For isomalt, 2 and 3 mm mini-tablets show better compactibility properties compared to 8 and 11.28 mm tablets especially at industrially relevant TS of around 2 MPa. The interpretation of 1 mm is more difficult, because the curve intersects the curves of 8 and 11.28 mm. At TS of 0.25 MPa, 2 MPa and 3 MPa the SF of the the tablets is lower compared to the SF of conventionally sized tablets. For a TS of 0.5 MPa the SF of 8 and 11.28 mm is lower than for 1 mm (0.75 to 0.85). We may assume from the course of the plot that with higher SF the TS could be higher for 1 mm tablets than for 8 and 11.28 mm. However, the scattering of CI_SF_ cannot be neglected, so the siginificance of the results is questionable.

The compactibility plots for Ludiflash® show interesting results regarding the compactibility profile of the tablets with different sizes. For TS between 1.5 and 2 MPa the 2 mm mini-tablets indicate superior compactibility, whereas with increasing SF no clear differences can be seen between all the other tablet sizes. Suprisingly, between a TS of 0.5 and 1.5 MPa the mean SF of 2 mm mini-tablets decreases. On the contrary, the CI_SF_ are larger for 2 mm mini-tablets between a SF of 0.7 and 0.8. Regarding the 3 mm mini-tablets a SF of almost 1 is reached with a TS of about 2 MPa, whereas conventionally sized tablets show higher TS at lower SF. For SF smaller than 0.9 higher TS are obtained with 3 mm tablets compared to conventionally sized tablets. According to previous patterns of 1 mm tablets the compactibility profile is not completely understood as non-significant results are obtained between 1 mm mini-tablets and conventionally sized tablets.

The compactibility of powders can be improved to a certain extent with mini-tableting for all different excipients and do support the previous observations of higher mechanical stability of mini-tablets with decreasing size (Lennartz and Mielck, 1998) It has to be pointed out, that the obtained data were generated with focusing on tableting at the same pressure and not at the same SF, which could be an explanation for the high scattering of CI_SF_ especially for

1 mm mini-tablets. Nevetheless, the results indicate higher compactibility for 2 and 3 mm mini-tablets almost independently from the used excipients at industrially relevant TS of 1–2 MPa (Leane et al., 2015; Sun et al., 2009).

### 3.4 Tablet dimensions and their effect on tabletability

**Fig. 5:**
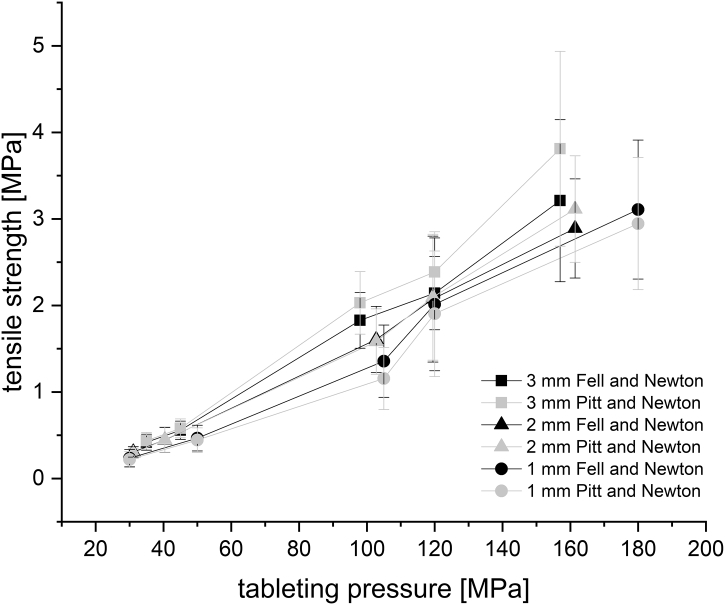
Tabletability plot of isomalt mini-tablets (1,2 and 3 mm); n = 10, mean ± SD.

**Fig. 6:**
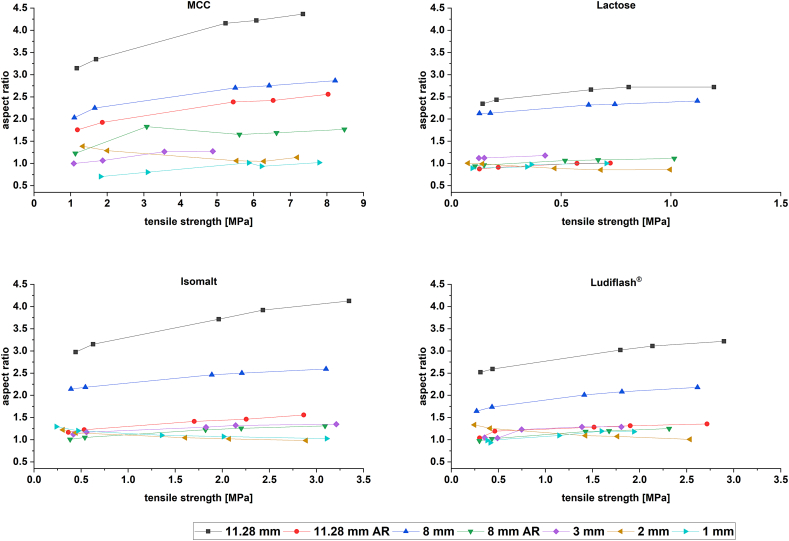
Plot of tensile strengths and aspect ratio (AR) for MCC, lactose, isomalt and Ludiflash® for different tablet sizes; n = 10, mean ± CI (95%).

The tensile strengths of all mini-tablets were calculated with the equation of Pitt et al. for convex tablets and with the equation of Fell and Newton for flat faced tablets (Fell and Newton, 1970; Pitt and Newton, 1988). The obtained tensile strengths are not significantly different for all excipients. The results are shown exemplarily for isomalt in Fig. 5. For simplification and standardisation, all plots were created using the equation of Fell and Newton (Fig. 6, Fig. 8).

The adjustment of aspect ratio (AR) for 8 and 11.28 mm tablets towards 1 does not lead to different TS compared to the original AR of the tablets for all excipients (Fig.6). Moreover, by increasing the TS, the AR of the conventional tablets changes, obviously due to the fact that the tablet height during tableting process is decreased. Compared to mini-tablets the AR changes slightly at different TS but remains in the area of about 1 to maximal 1.5 for all excipients. The masses of the tablets were of course not the same. As the focus of the study is prior to compare different dimensions within excipients with different properties and not the excipients witch each other, the masses are not adjusted (Table 3), due to different dosage heights, compressibility properties and densities of the used excipients.

**Table 3:** Masses (m) in mg of different excipients for different tablet sizes and adjusted aspect ratios (AR); mean ± SD, *n* = 10 for 8 and 11.28 mm tablets; *n* = 20 for 1–3 mm mini-tablets.

Tablet size [mm]	MCC		Lactose		Isomalt		Ludiflash®	
	m	SD	m	SD	m	SD	m	SD
1	0.9	0.1	0.9	0.1	0.9	0.2	0.7	0.0
2	7.2	0.1	5.6	0.2	6.5	0.2	6.1	0.9
3	13.7	0.4	16.1	1.0	15.0	0.5	15.6	1.2
8	187.5	1.3	219.2	1.7	197.4	1.3	236.8	2.0
8 AR	306.9	4.1	476.4	1.8	397.2	2.2	406.0	2.6
11.28	344.3	1.7	532.8	2.5	365.6	3.9	451.4	1.8
11.28 AR	594.0	18.5	1390.0	118.1	959.8	7.8	1055.0	6.1

For MCC, the changes of AR for conventionally sized tablets with increasing TS (and obviously tableting pressure) are the highest, as volume reduction leads to a reduced height of the tablet at a constant mass of the tablets. This effect is not distinctive for MCC mini-tablets. In contrast, lactose shows no big changes in AR with increasing TS for 8 mm AR and 11.28 mm AR tablets, but are still in the range of the original mini-tablets. The same effect is observed for isomalt and Ludiflash®. The tableting process at comparable pressures does not lead to a decrease in TS for tablets with approximated AR, but in fact shows TS highly comparable to non-approximated conventionally sized tablets.

**Fig. 7:**
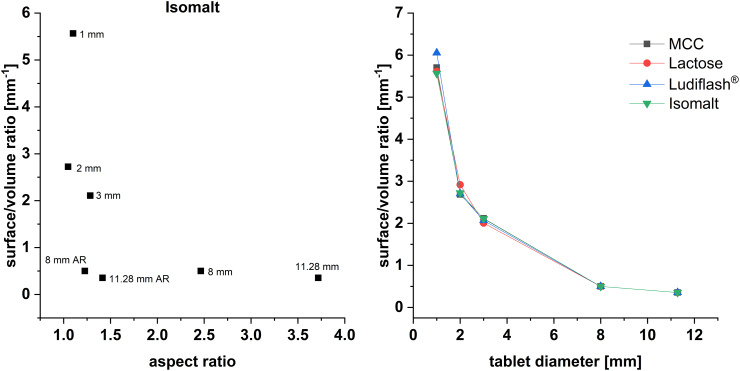
Impact on aspect ratio (left) on volume to surface ratio for isomalt tablets and conventional sized tablets with approximated aspect ratios (AR) and impact on tablet diameter (right) on volume to surface ratio for MCC, lactose, isomalt and Ludiflash® including conventional tablet with approximated aspect ratios.

The ratio between tablet surface and tablet volume, which is one of the main differences of mini-tablets compared to larger tablets, was not impacted by any of the excipients (Fig.7). The surface/volume ratio (SVR) of isomalt shows exemplarily that an adjustment of AR does not necessarily mean a change of SVR. This finding applies to all excipients and tablet sizes. Moreover, the plot reveals a second approach of defining mini-tablets besides the tablet diameter. A minimum SVR of 2 mm^−1^ represents a limit for mini-tablets. As the AR did not have an impact on TS and difference in tabletability between conventionally sized tablets and approximated conventionally sized tablets, the following plots neglect the data for AR tablets for a better visualisation.

For evaluation of the tabletability, the calculated TS is plotted against the tableting pressure. Two criteria can be used to analyse the quality of the tabletability. The first one is to evaluate the tableting pressure, where recommended suitable tensile strengths are in the range between 1 and 2 MPa (Leane et al., 2015; Sun et al., 2009). A second approach is the evaluation of the slope of the tabletability plots. The higher the slope, the better the tabletability properties are.

**Fig. 8:**
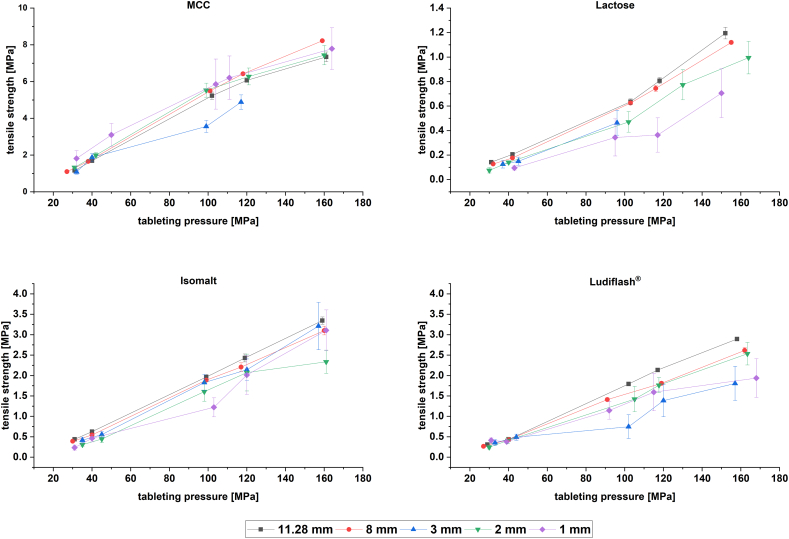
Tabletability plots of MCC, lactose, isomalt and Ludiflash® at different tablet sizes; n = 10; mean ± CI (95% confidence interval).

MCC shows the best tableting properties, followed by isomalt and Ludiflash®, whereas lactose shows the worst (Fig. 8). The tabletability plots do not show clear results regarding the impact of the diameter on the tabletability of the excipients. Among all excipients, MCC shows the best tableting properties, since even at low pressures high tensile strengths could be obtained for all tablet sizes due to its plasticity (Section 3.1.). The TS of 3 mm mini-tablets compressed at 160 MPa could not be measured due to overload limits of Texture Analyser. The effect of a plastically deforming material on tensile strengths, respectively tabletability as a quality attribute can also be seen for isomalt and Ludiflash®. Ludiflash® and isomalt show comparable tabletability plots. For both, proposed TS of 1–2 MPa are already reached at tableting pressures between 80 and 120 MPa. The brittle properties of lactose leading consequently to mainly mechanical bonding due to fragmentation and not plastic deformation, indicate the poorest tabletability properties. TS above 1 MPa are only reached at the highest tableting pressure. Furthermore, 3 mm tablets were not feasible to produce as high ejection forces over 1 kN were reached, despite of a sufficient external lubrication. Maybe a shift to internal lubrication would lead to lower ejection forces, but as the effect of the lubricant on the tablet properties should be excluded, this was not tested in the present study.

The tabletability plots reveal that in just one case the mini-tablets show better tableting properties compared to the 8 and 11.28 mm tablets, as 1 mm mini-tablets of MCC seem to be significantly better tabletable compared to the 11.28 mm tablets at lower tableting pressures. In fact, depending on the tableting pressure and excipient, some of the mean TS are significantly higher for the mini-tablets compared to the bigger sized tablets. However, the relevance of these differences is questionable.

To determine the effect of the tablet size on tabletability, a linear regression of the obtained tabletability plots is conducted (Table 4). The values were multiplied by 10^3^ for a better accessibility. For all excipients, the best tabletability properties are obtained by conventionally sized tablets, followed by mini-tablets. Just for isomalt, the 3 mm mini-tablets show a better tabletability according to the slope compared to 8 mm. But as the results are not significantly different, no clear statement can be made and just a trend can be described.

**Table 4:** Slope of linear regression of tabletability plots for MCC, lactose, isomalt and Ludiflash® presented as slope x 10^3^, *n* = 5; mean ± standard error of slope (SE); lower 95% confidence limit (LCL) and upper 95% confidence limit (UCL).

Tablet size [mm]	Slope	SE	LCL _95%_	UCL _95%_	Slope	SE	LCL _95%_	UCL _95%_
	MCC				Lactose			
11.28	49.4	3.6	37.9	60.9	8.4	0.6	6.4	10.5
8	55.5	2.2	48.5	62.6	8.0	0.4	6.8	9.2
3	39.5	5.4	16.5	62.5	5.9	0.4	1.3	10.4
2	49.2	4.6	34.5	63.9	6.8	0.5	5.4	8.3
1	45.7	4.4	31.6	59.8	5.4	1.1	0.6	10.1

	Isomalt				Ludiflash®			
11.28	22.7	0.1	22.4	23.1	20.6	0.51	19.0	22.3
8	21.1	0.4	19.9	22.4	17.5	0.41	16.2	18.8
3	22.6	1.0	19.3	26.0	11.3	2.07	4.7	17.9
2	17.4	2.0	11.0	23.8	17.1	0.53	15.4	18.7
1	20.9	2.7	12.3	29.6	12.1	1.24	8.2	16.1

For the comparison and evaluation of the plots, it is necessary to point out the different shapes of the tablets, besides the different sizes, due to different punch geometries. The powder movement and the distribution of the tableting pressure during tableting can lead to non-homogenous density regions within the tablet, where stress is mainly concentrated, which may lead to significant mechanical instabilities. This effect has been shown for flat-faced and convex curved tablets of MCC in several studies. Significant changes of powder movement and density regions within the tablet were found. Especially convex tablets showed a higher variety and mechanical failures compared to flat faced tablets (Eiliazadeh et al., 2004; Sixsmith and McClueskey, 1981). This effect of convex and flat faced mini-tablets in comparison to bigger sized tablets has not been investigated so far. But it may play an important role for interpretation of the obtained data as different distribution patterns may occur for mini-tablets compared to conventionally sized tablets.

It is further important to point out, that for calculation of the tensile strength the fracture and break of the tablet during the conduction of the test has to be diametral (Fell and Newton, 1970). For instance, the diametral stress on mini-tablets did not always lead to a desired diametral fracture of the tablet. Particularly for MCC and Ludiflash® tablets the mini-tablets did not always break, but were squeezed sometimes first by the punch of Texture Analyser and broke afterwards, most notably for 1 mm mini-tablets, which resulted in high scattering. In comparison to MCC and Ludiflash®, lactose and especially isomalt mini-tablets showed clear breaks, when applying diametral stress onto the tablet. These observations underline that suitable methods for analyzing the breaking force have to be developed and standardized for mini-tablets. Besides, the comparison of two measurement methods for breaking force is critical, as no apparatus is currently available to equally determine breaking forces for all tablets.

## 4 Conclusion

This study evaluated the influence of pharmaceutically relevant materials on the mini-tableting process compared to conventionally sized tablets on different levels.

The results of deformation behaviour tested with *in-die* Heckel plot and modified Weibull function lead to similar statements. *In-die* Heckel plots reveal that in most cases mini-tablets have significantly higher yield pressures compared to conventionally sized tablets. Only 3 mm MCC tablets show the lowest yield pressures and indicate more plastic deformation. A systematic relation between 1, 2 and 3 mm mini-tablets and change in yield pressure could not be found. These observations are supported by the analysis of β and γ values using the modified Weibull function. A reduction of the tablet diameter did neither lead to an increase of plasticity of the materials when using a multi-tip for the production of mini-tablets nor lead to a conclusion of a systematic behaviour within the mini-tablet sizes. However, a spot check at 100 MPa with a single-tip showed that for all excipients β and γ values were obtained, which are highly comparable with the results for 8 and 11.28 mm tablets. Nevertheless, the tableting with the single-tips was conducted at slower speed and subsequently longer dwell time, therefore this result has to be re-checked in another study. The adjustment of aspect ratio (AR) of the conventionally sized tablets towards 1 did not lead to expressive changes of yield pressures. This is in contrast to results of modified Weibull function, where this adjustment led to large shifts to very low β and γ values indicating mainly plastic deformation.

The profiles of specific plastic energy (SPE) do not contradict the results of the Heckel plot and the modified Weibull function. For all excipients, the SPE showed an almost linear dependency between tableting pressure and SPE for materials made as mini-tablets and conventionally sized tablets. The hypothesis of a more plastic behaviour of materials manufactured by mini-tableting can be rejected according to our observations. Nevertheless, a spot check with single-tip tooling revealed that there might be an impact of the tooling system for mini-tablets, as for identical tableting conditions (same dosage height and applied pressure) higher SPE was obtained for MCC and isomalt. However, the effect of mini-tablet tooling should be evaluated in a separate study.

Compactibility profiles show better compactibility for mini-tablets as in most cases higher tensile strengths (TS) were reached at given solid fraction (SF) of the tablet. It has to be noted, that all excipients were tableted at comparable tableting pressures and not targeting same SF. The purpose is that under industrial conditions tableting is performed aiming at certain TS by varying the tableting pressure, where a certain SF results from.

The effect of AR on tabletability showed that TS of conventionally sized tablets was not affected, as non-significantly different TS were obtained. Furthermore, it was shown, that just by adjusting the AR towards to mini-tablets did not lead to an adjustment of main characteristics of mini-tablets. Besides the defined size for mini-tablets, the surface/volume ratio (SVR) was found as a further characteristic property for mini-tablets, which cannot be impacted by changing tablet dimension of conventionally sized tablets.

Tabletability plots show that industrially relevant TS of 1–2 MPa are reached for MCC, isomalt and Ludiflash® at comparable low tableting pressures with all tablet sizes. Only for lactose higher tableting pressures are required due to the brittleness of the material. Regarding the slopes of the plots after a linear regression, mini-tablets did not show significantly superior tabletability properties.

## Declaration of Competing Interest

None.
